# Driving Online Healthcare Growth Amid the Digital Divide: How Trust in Professional Signals from Doctor Biographies Shapes Patient Decisions

**DOI:** 10.3390/healthcare13121418

**Published:** 2025-06-13

**Authors:** Hongyang Wang, Jian Jin, Li Li, Jiaqi Liu, Da Wang

**Affiliations:** 1 School of Information Technology and Management, University of International Business and Economics, Beijing 100029, China; 2 School of Government, Beijing Normal University, Beijing 100875, China; 3 School of International Trade and Economics, University of International Business and Economics, Beijing 100029, China; 4 School of Economics, Beijing International Studies University, Beijing 100024, China

**Keywords:** online healthcare, doctor biographies, patient trust, sentiment, consultation

## Abstract

Objectives: Online healthcare offers an effective solution to reduce regional disparities in medical access. However, building patient trust in a virtual environment, particularly amid digital divide challenges, remains critical for the sustainable development of healthcare platforms. This study investigates how doctors’ professional experience, communicated through online biographies, influences patient consultation decisions, aiming to uncover strategies that enhance trust and facilitate efficient doctor–patient matching. Methods: Drawing on trust theory and social distance theory, we develop an empirical model incorporating professional signals, follower community engagement, and sentiment intensity. Using text data and topic modeling from a leading online health platform, we analyze the impact of these factors on patient consultation behavior. Results: The findings demonstrate that professional experience significantly increases consultation purchases, partially mediated by active follower communities. Additionally, positive emotional expressions in biographies reduce perceived social distance, thereby strengthening trust and willingness to consult. These results highlight the combined effectiveness of professional signals and emotional cues in fostering patient trust. Conclusions: Strategically designed doctor profiles, integrating professional and emotional elements, can bridge the digital divide in online healthcare by enhancing trust and improving doctor-patient matching. This study advances the understanding of how online biographical narratives shape trust and decision-making, offering novel insights into the interplay of doctor-generated content, trust, and social distance.

## 1. Introduction

In recent years, online healthcare has received increasing attention for its potential to reduce regional disparities in medical resource distribution [1,2,3]. However, as patients and doctors are separated by a virtual environment, challenges such as trust deficits [4,5] and inefficiencies [6] have become more pronounced. Addressing these issues is critical not only for enhancing user experience and service quality but also for ensuring the development of online healthcare platforms [7]. Building trust and improving system efficiency help bridge the distance between providers and patients, ultimately fostering long-term engagement within online health ecosystems.

Although some studies have demonstrated a strong association between user-generated content (UGC) and the development of patient trust in online healthcare contexts, existing research has predominantly focused on patient-generated content, such as reviews [8,9] and ratings [10]. In contrast, the role of doctor-generated content in fostering trust and reducing digital divide remains underexamined. Moreover, existing studies often rely on behavioral data [11], overlooking the rich textual signals embedded in doctor-generated content. To address this gap, the present study applies text mining techniques to extract thematic and emotional cues from doctors’ biographies—one of the first and most salient pieces of information patients encounter when choosing a provider [12].

A doctor’s personal homepage functions as a vital information portal for online healthcare platforms, typically featuring details such as work experience, specialized expertise, educational background, research accomplishments, and patient reviews. These elements enhance doctors’ professional visibility, allowing patients to assess their qualifications more intuitively and thereby increasing the likelihood of consultation [13,14]. By promoting trust and efficient matching, doctor biography displays create mutual benefits for patients, doctors, and platforms, ensuring long-term platform development [9]. Encouraging doctors to enrich their personal homepages not only facilitates patients in finding suitable medical professionals but also boosts user retention and engagement, further driving platform activity. There is growing evidence that physicians’ professional backgrounds and online reputations play a critical role in improving outcomes in digital healthcare [7]. Although prior literature has examined the impact of UGC on patient decisions from various angles—such as physician characteristics [15,16], information quality [1,7], and patient perceptions [17]—limited attention has been paid to the professional information presented on doctors’ personal homepages. In online settings, where patients lack direct interaction with doctors, the issue of information asymmetry becomes even more pronounced [10,18].

As a result, professional information disclosed on doctors’ personal homepages serves as a key reference point for patients to evaluate a doctor’s qualifications, experience, and competence, thereby significantly strengthening patient trust [19]. Trust, in this context, is a crucial mechanism for mitigating uncertainty and perceived risk in online medical interactions. Many patients rely on doctors’ personal homepage content to form initial impressions and establish trust before initiating consultations [20]. However, there remains a lack of in-depth research explaining how trust in these personal homepages is developed.

Another notable characteristic of doctors’ homepages is the implicit expression of doctor–patient social distance within the conveyed information, which highlights the emotional dimension of the interaction. Social distance refers to the perceived disparity between individuals in terms of social attributes and cognitive perspectives, which directly influences the quality of interpersonal interactions [21]. It serves as a crucial lens for understanding patient psychology and decision-making processes. The perception of social distance influences how individuals interpret information and the extent to which they place trust in others [22]. Trust is less likely to develop naturally in contexts characterized by high social distance. In contrast, closer social distance fosters affect-based trust, a form of trust grounded in emotional connection, which can extend to others within the same social context.

High social distance undermines doctor–patient rapport, impairs communication, and reduces patient confidence in medical interactions [14]. This issue is especially prominent when patients lack medical knowledge, leading to distrust and uncertainty [23,24]. Research suggests that addressing emotional factors can reduce perceived social distance, reshape perceptions of proximity, and influence behavior [25,26]. Emotions not only affect how patients interpret interactions but also alter their perceived psychological distance from doctors, enhancing receptiveness to communication. Therefore, this study adopts social distance theory to examine patients’ psychological responses in online healthcare contexts [14,22,23].

This study analyzes the online biographies of 10,000 doctors from five major cities in China, confirming the significant impact of professional experience and follower engagement. The findings reinforce prior evidence that knowledge sharing plays a critical role in shaping patient behavior [27]. The results demonstrate that doctors’ professional experience not only fosters patient trust but also supports the development of follower communities, which in turn influence patients’ willingness to pay for medical consultation.

Critical medical decision-making necessitates physician supervision, particularly in scenarios requiring the integration of multimodal data, ethical considerations, and nuanced clinical judgment that current AI systems still require substantial improvement. Despite growing attention to online healthcare platforms, two critical gaps remain: (1) existing studies predominantly focus on patient-generated content (e.g., reviews) while neglecting how doctor-generated professional signals build trust [28,29]; (2) the emotional dimension of doctor profiles—a potential key to reducing social distance—has not been systematically examined through text mining approaches [21,22]. To address these gaps, this study investigates how the interplay between professional experience (as a competence signal) and sentiment intensity (as a social distance reducer) jointly shape patient decisions, with particular attention to the mediating role of follower communities. The findings are expected to advance our understanding of trust formation in digitally divided healthcare environments.

## 2. Literature Review

### 2.1. Patient-Generated Content and Doctor-Generated Content

While extensive research has explored UGC in online healthcare communities [8,30,31], most studies have primarily focused on patient-generated content, with limited attention paid to doctor part [15,32]. UGC broadly refers to information voluntarily created by users on digital platforms [33].

Research on patient-generated content has primarily focused on online comments made by patients [10,34]. Studies have shown that patient-generated UGC—such as reviews, comments [8], and word-of-mouth [31]—can reduce information asymmetry [18], enhance service quality [35,36], and improve patient satisfaction by influencing decision-making and feedback loops. For example, Kordzadeh [8] argues that online reviews exhibit a high level of systematic bias, which may mislead potential patients and contradict the responsibility of healthcare service providers to act in the best interests of patients. This indicates that the consensus of other patients on treatment experiences can influence a patient’s perception of treatment outcomes [37]. Additionally, certain language and other features of patients on medical portal websites may be associated with user behavior [34]. Prior work has also further discovered that the narrative authenticity and coherence of user texts can impact user decision-making behavior [38]. To sum up, Existing research has also revealed the positive impact of UGC on patient satisfaction and online healthcare service outcomes [11,30].

However, despite these contributions, relatively few studies have examined the role of content created by doctors [10]. Doctor-generated content, such as professional profiles and knowledge-sharing articles, may play a distinct role in shaping trust and facilitating effective doctor–patient matching—an area that remains underexplored in the current literature.

Some studies have delved into the factors influencing UGC from doctor users [15,16,39,40]. These studies found that factors like a doctor’s professional level, experience, qualifications, appointment transparency, service fees, and response quality have moderating effects on doctor-generated content [15]. Through the exploration of doctor-generated content, researchers also discovered that information quality, emotional support, and source credibility significantly and positively impact patients’ adoption of medical information [39]. Other antecedents stemming from doctor-generated content include the vocal features of doctor voice [16], personal information display [41] and knowledge sharing [40].

Especially, we notice some research found that emotional language signals, including negative emotions and language style matching, are effective in influencing patients in both obtaining information and emotional support from online community [10,39]. This suggests that emotional cues embedded in user-generated content can play a meaningful role in shaping patient perceptions and behaviors. While existing studies have largely focused on patient-generated emotional expressions, the emotional characteristics of doctor-generated content remain underexplored.

While these studies on UGC have directly explored the impact of individual user-generated information on patient perception and behavior, there is a variation in the focus on UGC in online healthcare community. However, they all delve into hidden information within UGC to reveal user behavior, yet none has directly established an analytical model for the relationship between UGC and patient purchasing decisions. Therefore, further investigation into doctor-generated content in online healthcare community is needed to uncover the differences in returns resulting from doctors’ long-term efforts on their profile content [42].

### 2.2. Trust

Trust serves as a critical facilitator in healthcare provision, with increasingly prevalent appeals for trust restoration in healthcare contexts [7]. Trust theory categorizes trust into two main types: interpersonal trust, built on emotional bonds and personal familiarity, and system trust, grounded in institutional mechanisms such as regulations and safeguards [43]. In online environments, trust plays a crucial role in reducing uncertainty and complexity in service interactions. Specifically, patients’ perception of a provider’s credibility and reputation significantly shapes their willingness to engage in online healthcare services [44]. Results from an online survey indicate that trust is the most critical factor in patients’ selection of online healthcare services [45]. The broader goals of trust are to create positive impressions, ensure confidence in the reliability of the provider, and provide a sense of security during service usage or transactions.

Nowadays, online consumers increasingly rely on user-generated content (UGC) when making purchase decisions. Once trust in a service or product is established through UGC, they are more likely to proceed with the transaction [46]. For example, potential customers in e-commerce are more inclined to trust the opinions of previous users, who share their experiences through reviews to inform and influence others’ purchasing decisions. In online healthcare environment, the knowledge shared by doctors also influences patients’ behavioral decisions by mitigating information asymmetry and fostering greater trust in the medical provider [15,40].

Trust plays a crucial role in how UGC influences decision-making, as it is built through real-life experiences shared by authentic users, helping others better understand and evaluate specific products or services [47,48]. We follow the widely accepted view that trust is a critical factor in the functioning and success of online healthcare communities [20,49], especially in light of the ongoing challenge of information asymmetry between online healthcare service providers and patients [50,51]. Therefore, we apply trust theory to elucidate how doctor-generated content contribute to trust formation in the context of consultation purchases.

### 2.3. Social Distance Theory

Social Distance Theory, as a critical theoretical framework for understanding interpersonal interactions and trust mechanisms, has been widely applied in fields such as social psychology, consumer behavior, and online interactions [21,22]. Social distance generally refers to the differences between individuals in terms of social attributes, status, psychological perceptions, or cultural backgrounds, which influence their understanding, trust, and the quality of their interactions [52]. According to the theory, the greater the perceived social distance, the lower the level of trust and willingness to interact between individuals. This phenomenon has been validated in various contexts, especially in virtual environments such as e-commerce [53] and online healthcare [54]. In online settings, the lack of face-to-face contact and the absence of real physical proximity amplify the sense of social distance between individuals.

The perception of social distance influences how individuals interpret information and the level of trust they place in others [22]. The greater the perceived social distance, the more abstract individuals’ perceptions of others tend to be, leading them to rely on overall impressions or stereotypes rather than understanding others through specific information. Consequently, in situations characterized by larger social distance, trust is less likely to develop naturally. In contrast, closer social distance fosters affective-based trust, which can extend to others within the same context.

When the social distance between patients and doctors is high, it becomes more difficult to establish rapport, provide information, and foster an understanding of the medical condition, which negatively impacts patients’ confidence in interacting with healthcare providers [14]. Further research has shown that a significant social distance often exists between doctors and patients, particularly when patients lack medical knowledge, which can lead to distrust and uncertainty [23,24].

However, one effective way to improve social distance is by focusing on individual emotions, as emotions can alter one’s perception of social distance and consequently influence behavior [25,26]. This suggests that by actively regulating emotions or managing emotional responses, it is possible to indirectly shape how patients engage in interactions. Emotions not only affect how patients interpret social situations but can also change their subjective perception of their distance from doctors, making it easier for patients to accept and engage with them. Therefore, this study adopts social distance theory as an additional perspective for understanding patients’ psychological responses [14,22,23].

## 3. Hypotheses Development

The personal information displayed on doctor biographies serves as an internal signal, distinct from the standardized platform information, and plays a crucial role in patients’ decision-making processes [55]. Platforms typically provide basic information about doctors, such as hospital affiliation and credential verification, which are external signals controlled and published by the platform [19,56,57]. In contrast, the exclusive information displayed on a doctor’s personal homepage, such as their professional background, years of experience, medical expertise, and training history, constitutes more individualized internal signals. This type of information not only showcases the doctor’s professional qualifications but also conveys their unique style, allowing patients to form a more personalized and specific impression of the doctor, beyond the standardized external signals provided by the platform. Such differentiated internal signals are particularly appealing to patients, as they enable a more intuitive understanding of the doctor, thereby enhancing the specificity and satisfaction of their choice [58].

These signals from doctors typically create a positive signal reception effect in the minds of patients, enhancing their attitudes and inclinations toward the doctor [57]. Compared to information provided uniformly by the platform, the content displayed on a doctor’s personal homepage tends to present more detailed insights into personal values and professional characteristics, thereby triggering trust and attention from patients. For example, when a doctor elaborates on their medical practice, specialized research areas, and treatment style on their personal homepage, patients are more likely to perceive the doctor’s attentiveness and professionalism, which further strengthens their perception and recognition of the doctor’s capabilities. Moreover, signal theory suggests that the accurate transmission of signals reduces the uncertainty caused by information asymmetry. Thus, these personalized internal signals create a bridge of trust between the doctor and the patient, helping to alleviate concerns arising from insufficient information. This, in turn, increases the patient’s positive regard for the doctor.

Particularly, the professional experience displayed on the doctor’s homepage, as a core factor, is the primary focus of patient attention and is crucial for building trust [58]. The enhancement of trust often directly influences patients’ purchasing decisions, as patients tend to choose the consultation services provided by doctors they perceive as more trustworthy. Therefore, it can be expected that the presentation of professional experience on the doctor’s homepage will have a positive impact on patients’ decisions to purchase consultation services. Based on this, this study proposes the following hypothesis:

**H1:**  *The professional experience displayed on the doctor’s personal homepage will have a positive impact on patients’ decisions to purchase consultation services.*

The sentiment intensity of the text in a doctor’s personal biographies helps reduce the social distance between the patient and the doctor, making it easier for patients to feel an emotional connection with the doctor [25]. This connection stems from the affinity effect of emotional expression on potential patients. When doctors convey positive and enthusiastic emotions through their personal biographies, patients are more likely to feel that the doctor is closer to them, thereby reducing the sense of unfamiliarity and distance. In medical services, particularly on online platforms where there is no face-to-face interaction, the emotions conveyed in the text are especially important. The increased sentiment intensity leads patients to develop a more intimate emotional experience while reading the doctor’s biographies, thus reducing social distance.

A smaller social distance typically increases trust, as people are more likely to trust those with whom they feel an emotional connection or psychological closeness. The smaller the social distance, the easier it is for patients to develop a sense of security and trust, further enhancing their willingness to interact with the doctor. On the other hand, when social distance is larger, individuals may become hesitant and cautious, which reduces the level of interaction and trust [53]. This is particularly true in medical contexts, where patients are at a disadvantage due to the information asymmetry caused by their lack of medical knowledge [59]. In such cases, patients are more likely to have a positive emotional response to the doctor. The reduction in social distance can help bridge this gap to some extent, allowing patients to build a foundation of trust even without fully understanding the doctor’s professional background.

Therefore, when a doctor’s personal biographies utilize a higher level of sentiment intensity in the text, patients may feel that the sense of distance between themselves and the doctor is reduced, especially when patients lack medical knowledge. This will help strengthen their trust in the doctor. In this way, sentiment intensity not only conveys the doctor’s care for the patient but also boosts the patient’s confidence, making them more willing to accept the doctor’s services. Based on this, this study proposes the following hypothesis:

**H2:**  *The sentiment intensity of the text in the doctor’s personal biographies will enhance the positive impact of professional experience on patients’ decisions to purchase consultation services.*

A doctor’s professional experience typically signifies the accumulation of extensive knowledge and skills, as well as the establishment of an authoritative image in the medical field. This professional image effectively attracts patients’ attention, allowing the doctor to build broad influence and a trustworthy brand perception on online platforms [10,30,40]. Particularly in the context where patients increasingly rely on online resources for health consultations and doctor selection, doctors with rich professional experience are more likely to stand out in the online environment. Such an authoritative image not only attracts the attention of potential patients but also provides a solid foundation for the growth of the doctor’s follower community on online platforms, thereby strengthening their overall online influence.

Furthermore, the follower community can serve as an indirect display of a doctor’s professional experience, and this display can be quickly amplified on online platforms [20]. A highly active follower community is often seen as a direct indicator of a doctor’s professionalism and popularity. This visible numerical representation not only attracts patients’ attention but also indirectly increases the doctor’s online exposure. With the gathering of a large number of fans, the doctor’s online image becomes more prominent and appealing, creating a demonstration effect: when potential patients browse a doctor’s biographies, doctors with a higher number of fans are often perceived as more trustworthy [40]. This demonstration effect may lead patients to view doctors with active follower community as more professional and reliable, thereby increasing their willingness to purchase consultation services.

Finally, through the shaping of their personal professional image and the accumulation of influence, doctors gradually attract more fans, thereby gaining a stronger presence and interactivity within social networks. Therefore, a doctor’s follower community becomes a key intermediary factor that links professional experience with the purchase of consultation services, making doctors with active follower community more attractive to patients, thus enhancing patients’ purchase decision tendencies. Therefore, this study proposes the following hypothesis:

**H3:**  *The activity level of a doctor’s online follower community mediates the relationship between professional experience and consultation service purchase.*

**Figure 1:**
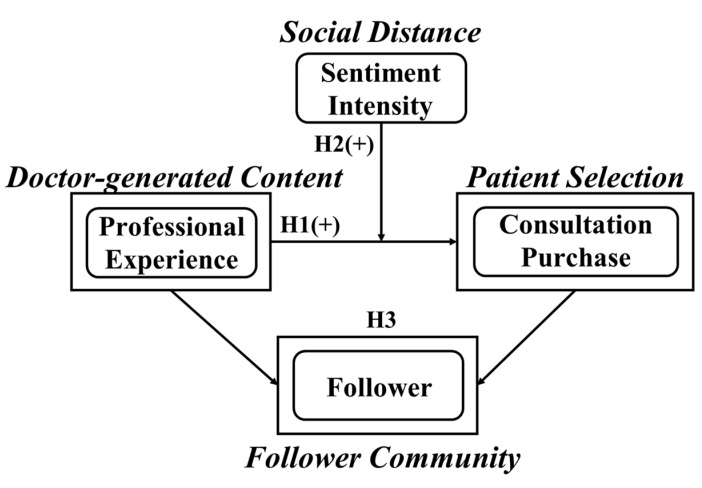
Research model.

The research hypothesis model constructed in this study is shown in Figure 1.

## 4. Methodology

### 4.1. Data Resources and Collection

Haodf.com is the largest medical website in China, covering nearly 500,000 doctors from over 6000 hospitals. It serves as a platform for Chinese patients to obtain medical consultations remotely from doctors, allowing them to provide comments or opinions on doctors from different regions. The consultation fees are determined independently by the doctors, with many adopting a fee-based model for their services. Typically, more renowned doctors charge higher fees for individual consultations.

Selecting Haodf.com for this study is justified by three reasons: (1) Representativeness: Haodf.com is the most widely used online healthcare platform in China, with millions of patients having received services on the platform, including online text consultations, phone consultations, outpatient appointments, and expert team consultations. Studying this platform is representative of the broader landscape. (2) Professionalism: Haodf.com is a specialized online healthcare service platform with over 610,000 doctors from more than 9900 different hospitals. Many of these doctors are from the highest professional-level hospitals in China. (3) Alignment with Research Objectives: Haodf.com openly displays authentic UGC and user behavioral records, providing reliable data support for our research.

Data were collected via a Python 3.7 program between September and October 2024. Initially, we identified the biographies of 10,000 doctors from five representative regions, Beijing, Shanghai, Shandong, Henan, Hubei, each with 2000 doctors. Subsequently, we gathered personal information, hospital details, doctor resume, and patient selection information from the doctors’ biographies. And the list of top-tier hospitals in each region were sourced from official Chinese government websites. Finally, we matched the hospitals based on the list of top-tier hospitals publicly available on the Chinese government’s website. This process resulted in a cross-sectional dataset comprising 10,000 samples.

### 4.2. Latent Dirichlet Allocation

Latent Dirichlet Allocation (LDA) is a widely used topic model in text mining and natural language processing [60]. It is a generative probabilistic model that assumes documents are composed of several topics, with each topic being represented by a distribution of words. LDA infers the topic distribution for each document and the word distribution for each topic by analyzing word frequency statistics.

The core formula of the LDA model is built through Bayesian inference. Suppose this study has D documents, a vocabulary size of V, and K topics. The goal of LDA is to find the following probability distributions:

P(θ|α)=Dirichlet(α)

where θ is the topic distribution for the document, and α is the hyperparameter representing the sparsity of the topic distribution.

P(ϕ|β)=Dirichlet(β)

where ϕ is the word distribution for the topic, and β is the hyperparameter representing the sparsity of the word distribution.

For each document:

Sample the topic distribution $\theta_{d}$ from the Dirichlet distribution Dir(α);

For each word in the document:

Sample a topic z from $\theta_{d}$;

Sample a word w from the word distribution Dir(β) of the corresponding topic z.

The generative probability for document d is:

$$P(w_{d}|\alpha ,\beta )=\int_{\theta_{d}}\;\int_{\phi }\;\prod_{n=1}^{N_{d}}\;P(w_{d,n}|z_{d,n},\phi )P(z_{d,n}|\theta_{d})P(\theta_{d}|\alpha )P(\phi |\beta )d\theta_{d}d\phi$$

Through Bayesian inference, this study ultimately obtains the topic distribution P(θ|w) for each document and the word distribution P(ϕ|w) for each topic.

### 4.3. Topic Extraction

During the data processing stage, it was necessary to preprocess the doctor profile texts to extract effective textual features for subsequent analysis. First, Chinese stop words were removed, and the text was segmented, breaking down the doctors’ self-introductions into individual words. Then, word frequency statistics and vectorization (bag-of-words model) were applied to retain the frequency information of words within the text.

**Figure 2:**
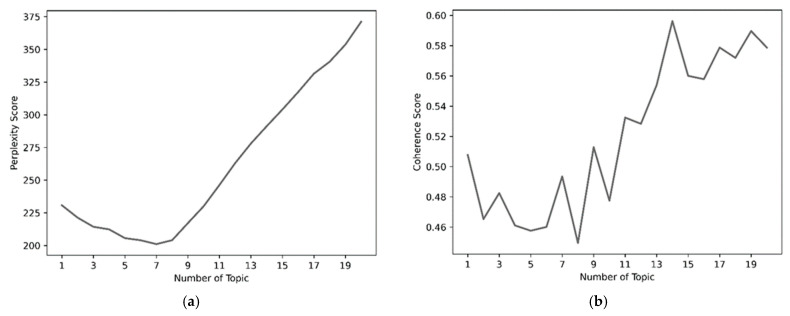
Indicators for topic partitioning. (**a**) Perplexity—topic number in LDA. (**b**) Coherence—topic number in LDA.

We applied the LDA model for topic extraction [61]. The number of topics in the model was determined based on a comprehensive evaluation of perplexity and coherence metrics. Setting the number of topics to nine resulted in relatively optimal performance in both perplexity and coherence, and thus, nine topics were ultimately selected to capture the multidimensional thematic structure of the texts (see Figure 2). During the training process, the LDA model iteratively inferred the word and topic distributions, continually optimizing its performance. With each iteration, perplexity decreased and coherence improved, allowing the final model to achieve an optimal balance between inter-topic distinctiveness and intra-topic consistency.

**Figure 3:**
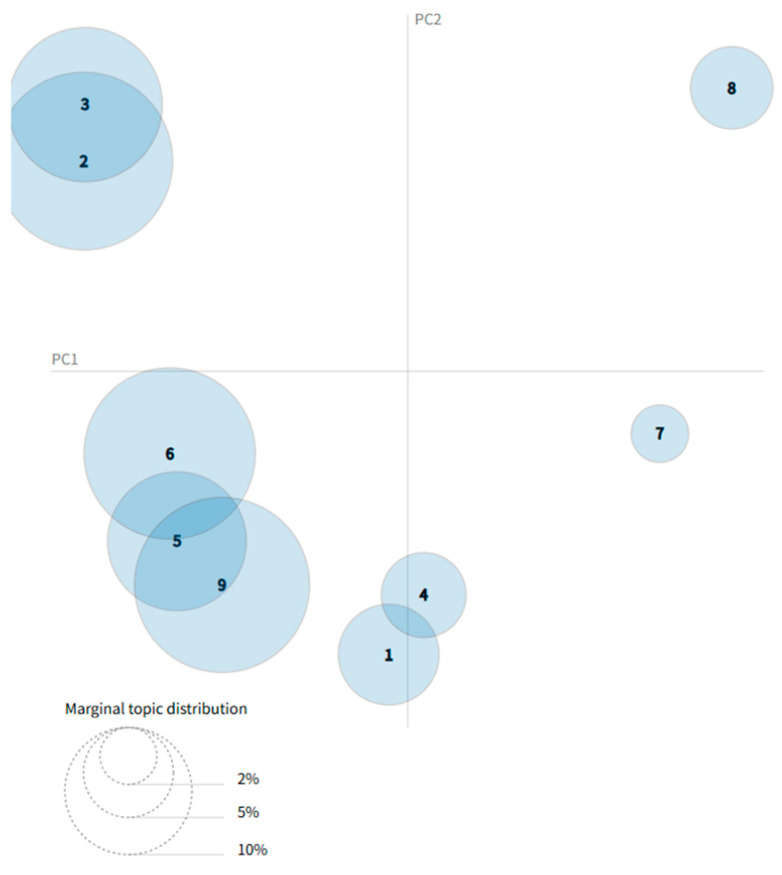
Topic visualization of doctor biographies [62,63].

We employed pyLDAvis [62,63] to generate a visualization of the topic clustering results from the physicians’ profile texts, as illustrated in Figure 3. In the visualization, each circle or bubble represents a distinct topic, with the size of the bubble indicating the prevalence of that topic within the corpus. The distance between bubbles reflects the degree of dissimilarity between topics—the greater the distance, the more semantically distinct the topics are; conversely, shorter distances suggest greater similarity or overlap. When two bubbles overlap, it indicates that the corresponding topics share common characteristics. For instance, the overlap between Topic 2 and Topic 3, as well as the intersections among Topics 5, 6, and 3, suggest a lexical or semantic overlap in their representative keywords.

After the topic extraction process, we assigned a primary topic label to each physician biography based on the distribution probabilities across the nine identified topics. During model training, each topic gradually developed a unique set of high-frequency keywords, which serve as the core descriptors of that topic. The probability distribution of these keywords within each topic not only indicates their relative importance but also reflects how they semantically define and differentiate one topic from another.

For example, in Topic 1, terms such as “Shanghai”, “China”, “membership”, “physician”, and “association” appeared with high probability, suggesting that these words are prominent within this topic and hold strong representative value. The LDA model leverages the distribution of such keywords to detect semantic signals within the text and categorize content accordingly. This process is carried out iteratively, with the model continuously refining the distribution of topic-specific keywords to enhance the clarity and interpretability of each topic.

Finally, the LDA model generated a probability distribution over the nine topics for each individual text, indicating the proportional contribution of each topic to that text. These weights, which sum to one, reflect the degree of association between the text and each topic. Based on these probabilities, the dominant topic for each text was identified as the one with the highest weight, which was then assigned as the text’s primary topic label. This label captures the central thematic focus of the text, while the remaining topic weights represent its secondary associations, thus preserving the thematic diversity within each document.

This approach enabled a clear and targeted categorization of each text into its most representative topic. The final results show that all texts were successfully categorized into the nine topics, with each topic defined by a set of representative keywords and a cluster of associated texts. This ensured that the topic classification was both accurate and practically useful. The distribution probabilities of the topic keywords are presented in Table 1 (only Topics 1, 4 and 7; for the full version, see Table A1 and Table A2).

### 4.4. Topic Aggregation

Following analysis of the extracted topics, this study found that some topics overlapped with each other and lacked clear differentiation. Therefore, it was necessary to screen and name the formed topics. We analyzed and named those topics with their characteristics:

Topic 1: Surgery and Plastic Surgery. Focuses on urological procedures and cosmetic surgeries (Key terms: urology, plastic surgery, cosmetology).

Topic 2: Medical Committees and Academic Activities. Reflects participation in professional associations and international collaborations (Key terms: Medical Association, international).

Topic 3: Positions in Medical Associations. Highlights leadership roles in professional organizations (Key terms: committee member, branch).

Topic 4: Orthopedic and Oral Reconstruction. Centers on trauma repair and reconstructive techniques (Key terms: orthopedics, stomatology, repair).

Topic 5: Tumor Treatment. Specializes in oncological surgical interventions (Key terms: tumor, treatment, surgery).

Topic 6: Clinical Career Development. Documents professional training and work history (Key terms: occupation, hospital, clinical).

Topic 7: Dermatological Treatments. Addresses medical dermatology and cosmetic procedures (Key terms: skin, dermatology, laser).

Topic 8: Academic Publications. Showcases research output in peer-reviewed journals (Key terms: journal, cancer).

Topic 9: Spine Surgery. Features minimally invasive spinal techniques (Key terms: spine, minimally invasive).

In the process of dividing the nine topics, the classification was primarily based on topic distance and semantic similarity of keywords. Although Topics 1, 4, and 7 maintain certain topic distance, they collectively contain primary terminology of medical departments, with particularly high keyword overlap in surgical procedures, repair, and cosmetic treatments. The specific classification basis is as follows:

1. Surgery and Surgical Operation (Topics 1, 4, 7): The keyword frequency in these three topics indicates they all involve surgeries and treatments in specific departments, such as urology, orthopedics, oral surgery, and dermatology. Despite differences in medical fields, they collectively focus on surgical procedures and repair treatments, and are thus categorized together.

2. Academic Roles and Committees (Topics 2, 3, 8): Keywords such as “committee”, “international journal”, and “professional association” frequently appear in Topics 2, 3, and 8, indicating these topics focus on doctors’ academic positions, research publications, and scholarly influence. Since these topics emphasize doctors’ academic backgrounds and committee memberships, they were merged into one category.

3. Tumor Treatment and Clinical Applications (Topics 5, 9): Topics 5 and 9 contain core terms like “tumor”, “surgical techniques”, and “minimally invasive”, primarily involving treatment methods for tumors and spine surgery, with strong similarities in surgical and technical applications. Both topics closely relate to clinical applications of tumor treatment and minimally invasive surgery, making them suitable for consolidation.

4. Career Development (Topic 6): Keywords in Topic 6 largely consist of “professional background”, “clinical experience”, etc., independently describing doctors’ career paths, educational backgrounds, and clinical experience. This topic lacks direct department or disease characteristic terms, mainly focusing on doctors’ professional growth, thus categorized separately as “Career Development” (see Table 2).

### 4.5. Data Processing

#### 4.5.1. Independent Variable

**Figure 4:**
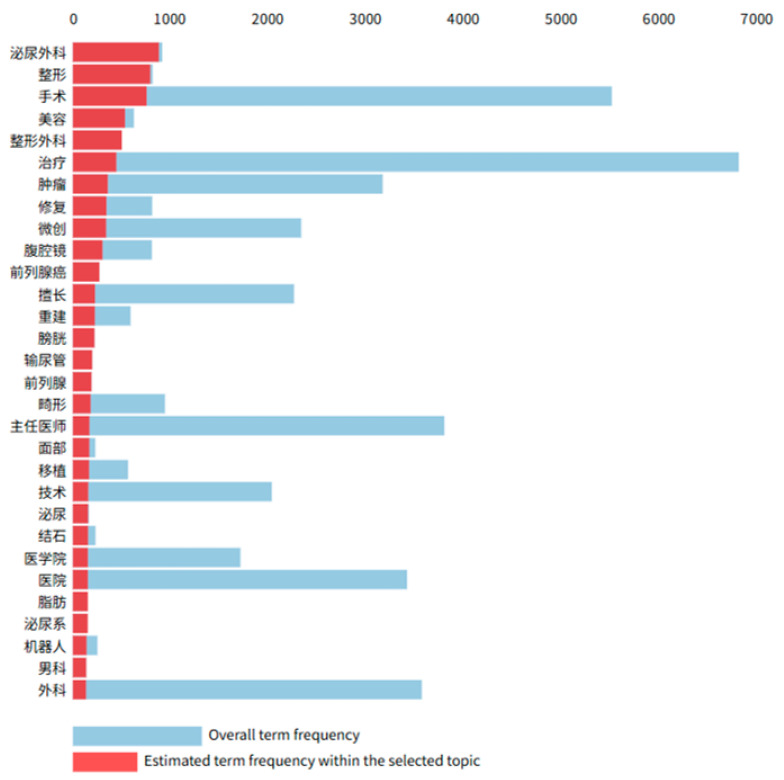
Keyword frequency of Topic 1 [63]. Note: 泌尿外科 = Urology, 整形 = Plastic Surgery, 手术 = Surgery, 美容 = Cosmetology, 整形外科 = Plastic Surgery, 治疗 = Treatment, 肿瘤 = Tumor, 修复 = Repair, 微创 = Microinvasive, 腹腔镜 = Laparoscopy, 前列腺癌 = Prostate Cancer, 擅长 = Specializes in, 重建 = Reconstruction, 膀胱 = Bladder, 输尿管 = Ureter, 前列腺 = Prostate, 畸形 = Deformity, 主任医师 = Chief Physician, 面部 = Facial, 移植 = Transplantation, 技术 = Technology, 泌尿 = Urology, 结石 = Calculus, 医学院 = Medical School, 医院 = Hospital, 脂肪 = Fat, 泌尿系 = Urinary System, 机器人 = Robot, 男科 = Andrology, 外科 = Surgery.

Topic 1 (see Figure 4) prominently features keywords related to specific surgical procedures, such as “urology”, “orthopedics”, “reconstruction”, “plastic surgery”, “aesthetic treatment”, and “therapy”. These keywords not only indicate the medical specialties that the physicians are engaged in but also reflect their accumulated expertise in surgical operations and related treatments. The surgical domain typically requires extensive hands-on experience to ensure the safety and effectiveness of procedures, encompassing a range of skills including patient evaluation, preoperative planning, surgical execution, and postoperative management.

The core message conveyed by these keywords is that the physicians possess advanced levels of professional competence and experience in specific surgical disciplines, beyond mere theoretical knowledge. Additionally, the frequent appearance of terms like “minimally invasive”, “reconstruction”, and “treatment” suggests a specialization in adopting newer, safer, and more efficient treatment modalities. This underscores not only technical proficiency but also a continuous pursuit of innovation and adaptability in clinical practice, highlighting the depth and breadth of their surgical experience.

Because the combined intensity of these surgery-related topics can serve as a proxy for a physician’s focus and accumulated experience in surgical procedures, this composite intensity (*Experience*) was used as an independent variable in the analysis. Specifically, the sum of topic intensities from Topics 1, 4, and 7 was employed to quantify a physician’s depth of expertise in fields such as urological surgery, plastic and reconstructive surgery, and aesthetic dermatology. The frequency and prominence of the keywords in these topics offer a meaningful measure of practical surgical experience and professional development.

#### 4.5.2. Dependent Variable

We designate patient consultation (*Consultation*) as the dependent variable, indicating which doctor a patient opts for in the online health community when seeking medical inquiries. On Haodf.com, each doctor’s individual profile exhibits the total count of online consultation services provided. Hence, we employ the doctor’s online consultation volume as a measure with which to assess patient preferences for a particular doctor.

#### 4.5.3. Moderating Variable

We define textual sentiment intensity (*Sentiment*) as the degree of emotional expression embedded in the language used on a doctor’s personal homepage, particularly within the self-introduction section. This measure reflects the implied social distance between the doctor and potential patients. A higher level of sentiment intensity indicates that the doctor communicates in a more warm, empathetic, and engaging manner, thereby conveying a lower perceived social distance.

As a moderating variable, sentiment intensity enables this study to further explore how social distance may shape patient trust and willingness to purchase consultation services. By highlighting the emotional tone of communication, it captures the subtle ways in which doctors’ language can foster psychological closeness and enhance the patient-doctor relationship in an online setting.

The interpretation of sentiment-related features (e.g., in doctors’ biographies) may vary significantly across cultural contexts. While our analysis focuses specifically on Chinese data, any generalization of these findings would require caution and further cross-cultural validation.

#### 4.5.4. Mediating Variable

We use the number of followers (*Follower*) on a doctor’s educational health account as the mediating variable to examine how the doctor’s social media influence impacts patient trust and their intention to purchase consultation services. Content shared through these accounts—such as medical education, health advice, and information on new medical technologies—plays a key role in shaping a professional and trustworthy image in the minds of patients.

As the number of followers increases, a doctor’s public influence also grows, suggesting that their content is receiving broad attention and recognition. Thus, follower count serves as an external indicator of the doctor’s influence. Doctors with a larger following are generally more likely to be trusted by patients, as wide audience approval is often perceived as a signal of strong professional competence and knowledge depth.

Table 3 presents the descriptive statistics for all variables. Variable processing follows the same procedures as in previous studies, with standardized *experience* for analysis.

The standardization procedure follows the formula:

$$Z=\frac{X-\mathrm{Mean}}{\mathrm{Std}.\;\mathrm{Dev}.}$$

where:

X is the original value;

Mean is the mean of the variable;

Std. Dev. is the standard deviation of the variable.

This transformation converts the variable into a z-score, indicating how many standard deviations a value is from the mean. Standardization ensures that variables are on a comparable scale, which is particularly important for regression analysis and interaction effect testing.

Table 4 presents the correlation coefficients among the variables. The results of the Pearson correlation analysis indicate that the correlation coefficient between consultation purchase (*Consultation*) and professional experience (*Experience*) is 0.067, which is significant at the 1% level, suggesting a weak positive correlation. The correlation coefficient between fans community (*Follower*) and professional experience is 0.074, also significant at the 1% level, indicating a weak positive relationship. The correlation coefficient between consultation purchase and sentiment intensity is −0.029, showing a weak negative correlation and reaching significance at the 5% level. The correlation between follower community and sentiment intensity (*Sentiment*) is −0.021, also indicating a weak negative correlation, but this result is not statistically significant. In addition, follower community is weakly correlated with hospital level (r = −0.033) and clinical title (r = 0.049), both displaying weak positive relationships. Overall, follower community appears to be an important factor influencing the purchase of consultation services, whereas the effect of sentiment intensity requires further investigation in subsequent analyses. The potential influence of control variables will also be examined through regression analysis.

To further test for multicollinearity among the variables, a Variance Inflation Factor (VIF) analysis was conducted. The results show that all VIF values are below 5, indicating the absence of multicollinearity issues, rendering the models reasonable and significant [64] (see Table 5).

### 4.6. Estimation

To test the research hypotheses, we employed ordinary least squares (OLS) regression and established the following multivariate regression model:

$$\begin{array}{cc} {\mathrm{Consultation}}_{i}= & \alpha_{0}+\alpha_{1}{\mathrm{Experience}}_{i}+\alpha_{2}{\mathrm{Experience}}_{i}\times {\mathrm{Sentiment}}_{i}+\alpha_{3}{\mathrm{Sentiment}}_{i}+\alpha_{4}{\mathrm{Hospital}}_{i}\\ & +\alpha_{5}{\mathrm{Appointment}}_{i}+\alpha_{6}{\mathrm{ClinicTitle}}_{i}+\alpha_{7}{\mathrm{Recommendation}}_{i}+\alpha_{8}{\mathrm{Ratings}}_{i}+\alpha_{9}{\mathrm{Price}}_{i}\\ & +\alpha_{10}{\mathrm{Duration}}_{i}+\alpha_{11}{\mathrm{Views}}_{i}+u_{i} \end{array}$$

where, Experience serves as the independent variable, Sentiment as the moderating variable. The subscript i indexes individual doctors. $\alpha_{0}$ denotes the intercept term, and $u_{i}$ captures the error term associated with observation i.

To further examine the mediating effect of the follower community, we employed stepwise regression analysis and developed the following multiple regression models:

$$\begin{array}{cc} \;\log({\mathrm{Consultation}}_{i}) & \\ & =\alpha_{0}+\alpha_{1}{\mathrm{Experience}}_{i}+\alpha_{2}{\mathrm{Hospital}}_{i}+\alpha_{3}{\mathrm{Appointment}}_{i}+\alpha_{4}{\mathrm{ClinicTitle}}_{i}+\alpha_{5}{\mathrm{Recommendation}}_{i}\\ & +\alpha_{6}{\mathrm{Ratings}}_{i}+\alpha_{7}{\mathrm{Price}}_{i}+\alpha_{8}{\mathrm{Duration}}_{i}+\alpha_{9}{\mathrm{Views}}_{i}+u_{i}\\ \;\log({\mathrm{Follower}}_{i}) & =\alpha_{0}+\alpha_{1}{\mathrm{Experience}}_{i}+\alpha_{2}{\mathrm{Hospital}}_{i}+\alpha_{3}{\mathrm{Appointment}}_{i}+\alpha_{4}{\mathrm{ClinicTitle}}_{i}+\alpha_{5}{\mathrm{Recommendation}}_{i}\\ & +\alpha_{6}{\mathrm{Ratings}}_{i}+\alpha_{7}{\mathrm{Price}}_{i}+\alpha_{8}{\mathrm{Duration}}_{i}+\alpha_{9}{\mathrm{Views}}_{i}+u_{i}\\ \;\log({\mathrm{Consultation}}_{i}) & \\ & =\alpha_{0}+\alpha_{1}{\mathrm{Experience}}_{i}+\alpha_{2}{\mathrm{Follower}}_{i}+\alpha_{3}{\mathrm{Hospital}}_{i}+\alpha_{4}{\mathrm{Appointment}}_{i}+\alpha_{5}{\mathrm{ClinicTitle}}_{i}\\ & +\alpha_{6}{\mathrm{Recommendation}}_{i}+\alpha_{7}{\mathrm{Ratings}}_{i}+\alpha_{8}{\mathrm{Price}}_{i}+\alpha_{9}{\mathrm{Duration}}_{i}+\alpha_{10}{\mathrm{Views}}_{i}+u_{i} \end{array}$$

The variable Follower was introduced into the model as a mediating variable. Besides, a logarithmic transformation was applied to the dependent variable to align its scale with that of the mediating variable, thereby facilitating more accurate regression estimation.

## 5. Empirical Results

### 5.1. Main Effect and Moderation Effect

To test the hypothesized relationships, we performed a series of regression analyses to examine how each variable influences the purchase of consultation services. Table 6 presents the empirical findings. Model 1 displays the results of the main effect, Model 2 incorporates the sentiment intensity to assess its direct effect, and Model 3 includes the interaction term between sentiment intensity and professional experience to explore potential moderation effects.

In Model 1, the coefficient of the key explanatory variable experience is 332.658 and is statistically significant at the 1% level, indicating that, with a high degree of confidence, physicians’ professional experience significantly increases patients’ willingness to purchase consultation services. This finding supports Hypothesis H1, which posits that greater professional experience enhances patients’ intention to purchase services.

In Model 2, after introducing the sentiment variable, the coefficient of experience is 321.643 and remains statistically significant at the 1% level. This indicates that the increase in professional experience continues to exert a significant and positive effect on consultation service purchases. The result is consistent with Model 1 and aligns with theoretical expectations.

Model 3 examines the interaction between sentiment and experience. The results show that the interaction term has a coefficient of 464.289 and is statistically significant at the 5% level, indicating that the effect of professional experience on consultation service purchases is significantly strengthened as sentiment intensity increases. This finding suggests that emotional factors in physicians’ profile pages may play a moderating role, making patients more inclined to purchase services when sentiment intensity is higher, thereby supporting Hypothesis H2.

**Figure 5:**
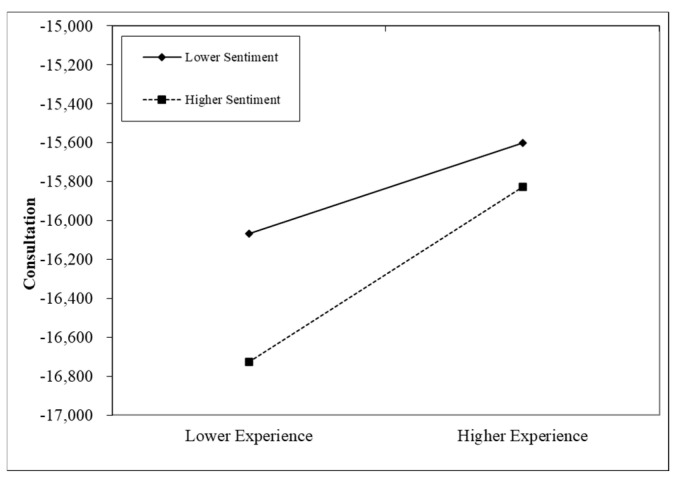
The moderating effect of sentiment intensity on professional experience.

Figure 5 illustrates the moderating effect of sentiment on the relationship between experience and consultation purchase. Following conventional practice, professional experience was categorized into high and low levels using one standard deviation above and below the mean, respectively. The figure presents the interaction patterns under low (solid line) and high (dashed line) levels of sentiment intensity. It can be observed that when sentiment intensity is high (dashed line), the positive effect of professional experience on consultation service purchase becomes significantly stronger. This suggests that as sentiment increases, the positive association between professional experience and consultation service purchase is amplified. One possible explanation is that physician biographies with higher sentiment intensity are more likely to elicit patient trust and positive emotions, thereby enhancing the perceived value of the physician’s professional background [27]. In other words, high sentiment intensity magnifies the impact of professional experience, making it more influential in shaping patients’ purchase decisions.

The above analysis indicates that professional experience alone may be insufficient to drive patients’ consultation purchase; rather, the positive expression of emotion can enhance the practical impact of professional experience. This finding carries important managerial implications for online healthcare platforms, suggesting that encouraging physicians to convey a warmer and more emotionally engaging tone in their self-descriptions may help increase the likelihood of purchases.

### 5.2. Mediating Effect of Follower Community

We employed stepwise regression [65] to test the mediating effect of follower community, using multivariate regression analysis conducted in STATA. It is worth noting that the stepwise regression approach is a widely adopted approach for variable selection due to its computational efficiency and intuitive implementation. While this method has proven valuable for exploratory analyses like ours, we acknowledge its limitations regarding sensitivity to data fluctuations and variable selection stability. Nevertheless, for our primary objective of identifying plausible predictors in this preliminary investigation, the approach provides sufficient utility.

Table 7 presents the results of the mediation analysis examining the impact of follower community on consultation service purchases. All models report adjusted R-squared values exceeding 0.30, and F-statistics above 150, indicating the models are robust and statistically valid. To assess multicollinearity, we conducted a Variance Inflation Factor (VIF) test. The VIF values for all independent variables are below 10, suggesting that multicollinearity is not a concern and the models are well specified [66].

Table 7 presents the empirical results of the mediating effect of follower community. Model 4 serves as the baseline for stepwise regression, illustrating the effects of professional experience, hospital level, appointment services, clinical title, and other variables on consultation service purchases. The regression coefficient of professional experience on consultation purchase is 0.136 and is statistically significant at the 1% level (*p* < 0.001). This indicates a significant positive effect of professional experience on consultation service purchases, satisfying the first condition for testing a mediation effect.

In Model 5, the follower community is treated as the dependent variable. The results reveal a significant positive association between the follower community and consultation purchase, with a coefficient of 0.877, significant at the 1% level. This suggests that professional experience positively influences the development of the follower community, indicating the potential presence of a mediating effect.

In Model 6, both professional experience and follower community are included to examine the impact of the follower community on consultation service purchases and to assess whether the direct effect of professional experience significantly diminishes or disappears. The results show that the regression coefficient for the follower community on consultation service purchases is 0.877, significant at the 1% level (*p* < 0.001), indicating a significant positive influence of the follower community on consultation service purchases. The coefficient for professional experience decreases from 0.136 in Model 4 to 0.031 in Model 6, with a reduction in significance, though it remains significant at the 5% level (*p* < 0.01). This suggests that the direct effect of professional experience has diminished.

Based on the above results, follower community plays a partial mediating role between professional experience and consultation purchase. Professional experience not only directly influences consultation service purchases but also indirectly enhances the willingness to purchase through the active follower community.

In Model 6, the inclusion of follower community significantly weakens the direct effect of professional experience on consultation service purchases, though it does not completely eliminate it. Therefore, it can be concluded that follower community partially mediates the relationship between professional experience and consultation service purchases, supporting Hypothesis H3 of this study.

### 5.3. Robustness Check

We conducted multiple robustness checks to ensure the reliability of the main effects and moderating effects, as presented in Table 8. Given that the length of text may potentially influence users’ comprehension of information and their purchase intention, we included *text length* as a control variable in the original model to assess its impact on other variables and thereby enhance the robustness of the regression results. In Models 7, 8, and 9, the key independent variables, including *Experience* and *Sentiment*, remained statistically significant and maintained consistent directions of association with purchase intention as observed in the baseline models, demonstrating the robustness of the findings. Moreover, the inclusion of text length did not substantially alter the magnitude or significance of the coefficients of the other variables. This suggests that text length does not confound the main effects. Accordingly, the results of the robustness checks confirm that the model specification is appropriate and that the regression findings offer stable and valid explanations of purchase behavior.

Table 9 presents the robustness checks for the mediating effect of the follower community. We employed two approaches to validate the robustness of the baseline regression results: adding the control variable “text length” and applying a negative binomial regression model. In the regression models that included text length, the effect of the follower community on consultation purchase remained statistically significant, and the coefficients of other variables changed only marginally. This indicates that the inclusion of text length did not materially affect the main results.

Furthermore, the relationship between the follower community and consultation purchase remained significant under the negative binomial regression, which further supports the robustness of the follower community’s mediating role in the model.

Across all robustness checks, the significance and direction of the key independent variables remained consistent, confirming the stability of the baseline findings. For example, in Models 14 and 15, the positive effect of the follower community on consultation purchase remains significant, with minimal variation in the coefficients of other variables and no evidence of substantial bias. These results suggest that the model is well specified and that the regression findings are both robust and reliable, unaffected by external variables or alternative modeling choices.

## 6. Discussion

### 6.1. Key Findings

This study primarily explored various factors influencing doctor’ online consultation service purchase, focusing on the interplay between doctors’ professional experience, follower community, and the sentiment intensity of their biographies. This study first hypothesizes that online follower community may serve as mediators between doctors’ professional experience and consultation service purchase intention. Doctors’ rich professional experience typically conveys an authoritative image, further attracting numerous followers, thereby enhancing the doctor’s visibility and trustworthiness in social networks. Building on this foundation, patients tend to trust doctors with highly active follower community when selecting doctors, which becomes an important factor driving consultation service purchase [10,30].

The study employs stepwise regression [65] to examine relationships between variables and conducts detailed exploration of model stability. Within the multiple regression models constructed, consultation service purchase intention serves as the dependent variable, while professional experience and follower community function as independent variables. Sentiment intensity acts as a moderating variable to analyze the impact of patients’ emotional attitudes toward doctors. Through the stepwise regression process, the significant effects of professional experience and follower community are progressively confirmed, revealing that sentiment intensity positively influences patients’ trust and willingness to select doctors [27]. These findings indicate that follower community not only reflect doctors’ online influence but also largely drive patients’ purchasing decisions through the demonstration of professional image.

Additionally, the study incorporates multiple control variables and employs negative binomial regression analysis to ensure the robustness of analytical results. After adding control variables such as text length, the results continue to support the hypothesized mediating effect, validating the model’s stability and explanatory power. The negative binomial regression results similarly indicate that doctors’ professional image and online influence significantly impact consultation service purchase, whether through the demonstration effect of active follower community or the positive influence of sentiment intensity. This research not only enriches understanding of doctors’ influence on online medical platforms but also provides empirical evidence for related platforms to optimize the display of generated content on doctors’ homepages and facilitate patient selection.

### 6.2. Practical Implications

This study helps online medical platforms optimize information display strategies on doctors’ homepages, particularly in highlighting doctors’ professional experience through LDA model to enhance patient trust and service purchase intention. While LDA modeling has inherent computational limitations for real-time applications, its strengths in interpretable topic modeling remain valuable for medical text analysis, particularly in non-time-sensitive clinical scenarios. Research indicates that the intensity of medical experience topics such as “surgery and surgical procedures” on doctors’ homepages significantly impacts patients’ decision-making. Therefore, platforms should prioritize displaying doctors’ professional skills, surgical experience, and high-value information directly related to medical services. This approach not only helps patients quickly understand doctors’ professional backgrounds but also effectively conveys signals that strengthen patients’ perception of and trust in doctors’ professionalism.

Furthermore, the platforms can utilize topic analysis and natural language processing technologies to automatically analyze and categorize content on doctors’ homepages, presenting structured information on topics of high patient interest. For example, platforms can establish sections such as “Specialized Treatment Areas”, “Typical Cases”, or “Surgical Outcomes”, allowing patients to intuitively access information relevant to their needs. Additionally, platforms can identify key topics influencing patient selection by analyzing patient feedback data and optimize recommendation mechanisms on doctors’ homepages accordingly, helping patients more efficiently match with suitable doctors.

### 6.3. Theoretical Implications

The rapid development of online healthcare reflects the continuously growing demand for information in the medical and health fields among users [11,30]. To more deeply understand doctor-generated content in online healthcare community, this research identifies hidden professional experience in doctor-generated content and examines its impact on consultation service purchases. Additionally, the study investigates the mediating effect of follower community. Our study contributes to the theoretical literature in several ways. First, by elucidating the importance of trust in doctor-generated content in online healthcare, we provide a nuanced insight into trust theory [43]. This research specifically examines how patient trust in doctors is influenced by doctors’ professional experience and follower community. Trust theory typically emphasizes that trust establishment relies on credible indicators such as professional skills and social recognition. In this context, doctors’ professional experience and follower community jointly form the foundation for patients’ judgment of doctor credibility. This provides new empirical support for trust theory, indicating that in online medical settings, patient trust stems not only from doctors’ professional authority but is also significantly influenced by doctors’ online influence and social recognition (such as follower community).

Second, by introducing social distance theory [21,22], this research reveals how sentiment intensity, as a variable measuring social distance, functions in doctor-patient interactions. Sentiment intensity reflects patients’ emotional tendencies toward doctors, significantly influencing patients’ perception of social distance and consequently altering their selection behavior. Social distance theory has traditionally been used to analyze how psychological and emotional distance between individuals affects behavior [53]. Building on this foundation, this study further demonstrates that in online contexts, positive emotional expression can shorten social distance between doctors and patients, helping to build patient trust and willingness to select [27]. This not only enriches the application scenarios of social distance theory but also expands its operational mechanisms in doctor-patient interactions.

Third, this research combines trust and social distance theories to analyze the comprehensive influence of sentiment intensity, professional experience, and follower community on patients’ purchase intention. The study finds that patients’ trust in doctors interacts with social distance, jointly affecting patients’ decision-making. This finding not only helps deepen understanding of the complex relationship between trust and social distance in online environments [53] but also provides a new perspective for integrating trust theory and social distance theory. On digital platforms, the interaction mechanism between trust and social distance exhibits characteristics different from offline settings, suggesting that in online environments, trust and emotional factors are more sensitive and direct, thereby influencing patients’ decision-making processes. Through these findings, this research to some extent fills the research gap regarding trust and social distance theories in digital health platforms.

## 7. Conclusions

This research explores how doctor biographies influence patients’ consultation purchases, using trust theory and social distance theory. Specifically, it investigates how doctors’ professional experience, online follower communities, and sentiment intensity affect patients’ purchase decision, with follower communities partially mediating the effect between professional experience and purchase intention. The findings show that doctors’ professional experience enhances patient trust, while the follower community mediates this effect. Additionally, sentiment intensity, as an indicator of social distance, reflects emotional attitudes in doctor-generated content, influencing patient preferences.

The practical significance of this study lies in its potential to help online healthcare platforms optimize the displays of doctor profiles, improve doctor-patient matching efficiency, and support platform development amid digital transformation. It offers valuable insights for enhancing patient trust and promoting the long-term development of online healthcare.

## Figures and Tables

**Table 1:** Probability distribution of topic keywords.

Topic 1		Topic 4		Topic 7	
Keywords	Distribution Probability	Keywords	Distribution Probability	Keywords	Distribution Probability
Urology	0.03	Orthopedics	0.033	Skin	0.022
Plastic	0.027	Stomatology	0.028	Dermatology	0.022
Surgery	0.026	Joint	0.023	Dermatology Department	0.021
Cosmetology	0.018	Surgery	0.018	Treatment	0.016
Plastic Surgery	0.017	Sports	0.018	Cosmetology	0.01
Treatment	0.015	Injury	0.016	Cicatrix	0.009
Tumor	0.012	Maxillofacial	0.016	Hemangioma	0.009
Repair	0.012	Trauma	0.015	Lupus Erythematosus	0.008
Microinvasive	0.012	Repair	0.015	Laser	0.008
Laparoscopy	0.01	Arthroscopy	0.011	Acne	0.008
Prostate Cancer	0.009	Fracture	0.011	Dermatovenereology	0.008
Specializes in	0.008	Treatment	0.01	Rheumatism	0.008
Reconstruction	0.008	Oral Medicine	0.01	Psoriasis	0.007
Bladder	0.008	Reconstruction	0.01	Research	0.007
Ureter	0.007	Knee Joint	0.009	Systemic	0.007
Prostate	0.007	Deformity	0.009	Hair	0.007
Deformity	0.006	Medical School	0.008	Specializes in	0.006
Chief Physician	0.006	Replacement	0.008	Burn	0.006
Facial	0.006	Chief Physician	0.007	Sexually Transmitted Disease	0.006
Transplantation	0.006	Implantation	0.007	Facial	0.006

Note: The raw text is in Chinese, and the results of word segmentation are slightly different from those of English word segmentation.

**Table 2:** Topic classification and aggregation.

Category	Topic Number	Topic Name	Topic Description
Surgery and Surgical Operation	1	Surgery and Plastic Surgery	Involves surgical procedures and treatment methods in urology and plastic surgery fields, including urinary system operations, plastic and cosmetic surgeries.
	4	Orthopedic and Oral Surgical Repair and Reconstruction	Primarily involves surgical repair and tissue reconstruction techniques in orthopedics and oral surgery, especially repair of fractures, joints, maxillofacial areas, etc.
	7	Skin Diseases and Cosmetic Treatments	Focuses on cosmetic and treatment methods in dermatology, involving content related to diagnosis and treatment of skin diseases and cosmetic repair.
Academic Roles and Committees	2	Medical Committees and Academic Activities	Reflects doctors’ positions in medical academic committees, participation in international academic activities and conferences, highlighting academic identity and industry status.
	3	Positions in Medical Professional Associations and Committees	Focuses on doctors’ identities and positions in medical professional associations, emphasizing doctors’ professional backgrounds and recognition in specialized fields.
	8	International Journal Publications and Academic Impact	Emphasizes doctors’ academic publications in international journals and the resulting academic influence, reflecting doctors’ international academic standing.
Tumor Treatment and Clinical Applications	5	Tumor Treatment and Surgical Techniques	Focuses on treatment in the tumor field and application of surgical techniques, including tumor resection, minimally invasive surgery, and related technologies.
	9	Spine and Minimally Invasive Surgical Applications	Focuses on spine surgery and application of minimally invasive techniques in surgery, including development and application of minimally invasive surgery in spine surgery.
Career Development	6	Career Development and Clinical Work Experience	Describes doctors’ career development experiences, including educational background, professional experience, workplace, and extensive clinical practice experience.

**Table 3:** Descriptive statistics of variables.

Variable	Mean	Std. Dev.	Min	Max	Definition
Consultation	3247.38	4941	0	64998	Cumulative number of patients the doctor has treated
Experience	0	1	−0.65	3.86	The sum of the standardized intensity scores of Topics 1, 4, and 7
Follower	3167.64	4448	1	55,000	Number of followers on the doctor’s account
Sentiment	0.92	0.23	0	1	Sentiment intensity score of the doctor’s personal profile text
Hospital	0.96	0.19	0	1	Whether the doctor is from a Grade A tertiary hospital (1 = yes, 0 = no)
Appointment	0.65	0.48	0	1	Whether appointment booking service is activated (1 = yes, 0 = no)
ClinicTitle	3.40	0.74	1	4	Clinical professional title level (1 = other, 2 = attending physician, 3 = associate chief physician, 4 = chief physician)
Recommendation	4.02	0.44	3.3	5	Recommendation score assigned to the doctor by the platform algorithm
Ratings	0.72	0.44	0.01	1	Average rating given to the doctor by users
Price	70.91	103.00	0	1500	Price for a single online consultation
Duration	3521.83	1322.80	12	5435	Cumulative number of days the doctor has been active on the platform
Views	208,963.20	814,112.40	0	32,046,000	Cumulative views of the doctor’s educational articles

**Table 4:** Correlation matrix of variables.

Variable	(1)	(2)	(3)	(4)	(5)	(6)	(7)	(8)	(9)	(10)	(11)	(12)
Consultation	1											
Experience	0.067 ***	1										
Follower	0.867 ***	0.074 ***	1									
Sentiment	−0.029 *	−0.071 ***	−0.021	1								
Hospital	−0.055 ***	0.021	−0.033 **	0.018	1							
Appointment	0.178 ***	0.021	0.204 ***	−0.006	−0.029 *	1						
ClinicTitle	0.103 ***	−0.174 ***	0.049 ***	0.117 ***	0.013	−0.043 ***	1					
Recommendation	0.427 ***	0.041 ***	0.515 ***	−0.001	−0.034 **	0.311 ***	−0.091 ***	1				
Ratings	0.274 ***	0.073 ***	0.313 ***	−0.066 ***	−0.091 ***	0.361 ***	−0.292 ***	0.567 ***	1			
Price	0.239 ***	−0.056 ***	0.321 ***	0.026 *	0.027 *	0.059 ***	0.147 ***	0.243 ***	0.162 ***	1		
Duration	0.246 ***	−0.036 **	0.148 ***	0.071 ***	0.034 **	−0.017	0.516 ***	−0.031 **	−0.177 ***	0.146 ***	1	
Views	0.335 ***	0.011	0.311 ***	0.005	−0.077 ***	0.067 ***	0.038 **	0.160 ***	0.084 ***	0.108 ***	0.130 ***	1

(1) = Consultation, (2) = Experience, (3) = Follower, (4) = Sentiment, (5) = Hospital, (6) = Appointment, (7) = ClinicTitle, (8) = Recommendation, (9) = Ratings, (10) = Price, (11) = Duration, (12) = Views. *** *p* < 0.01, ** *p* < 0.05, * *p* < 0.1.

**Table 5:** Multiple collinearity test.

Variable	Experience	Follower	Sentiment	Hospital	Appointment	Clinic-Title	Recommendation	Ratings	Price	Duration	Views	Mean VIF
VIF	1.05	1.61	1.02	1.02	1.15	1.52	1.82	1.65	1.17	1.43	1.12	1.32
1/VIF	0.95	0.62	0.98	0.98	0.87	0.66	0.55	0.60	0.86	0.70	0.89	-

**Table 6:** Standardized estimation of main and moderation effect.

Variable	Model (1)	Model (2)	Model (3)
	Consultation	Consultation	Consultation
Experience	332.658 *** (67.298)	321.643 ***(68.489)	−85.596 (198.088)
Experience * Sentiment	-	-	464.289 ** (222.696)
Sentiment	-	−889.938 ** (370.587)	−954.335 ** (383.363)
Hospital	−712.955 * (428.059)	−698.535 (428.544)	−687.838 (429.819)
Appointment	282.950 ** (124.558)	289.350 ** (124.614)	271.754 ** (124.491)
ClinicTitle	277.864 *** (110.322)	301.156 *** (110.121)	302.635 *** (110.142)
Recommendation	3618.376 *** (265.360)	3634.267 *** (266.028)	3635.505 *** (265.965)
Ratings	976.717 *** (150.707)	950.641 *** (150.985)	948.825 *** (151.185)
Price	4.379 *** (0.854)	4.395 *** (0.857)	4.406 *** (0.859)
Duration	0.824 *** (0.070)	0.827 *** (0.071)	0.825 *** (0.071)
Views	0.001 *** (0.000)	0.001 *** (0.000)	0.001 *** (0.000)
constant	−15,905.060 *** (1055.458)	−15,240.040 *** (1051.923)	−15,177.210 *** (1049.130)
Observations	4126	4125	4125
R-squared	0.326	0.328	0.328
F test	126.79	114.62	106.97

Robust t-statistics in parentheses. *** *p* < 0.01, ** *p* < 0.05, * *p* < 0.1.

**Table 7:** Standardized estimation of the mediating effect.

Variable	Model (4)	Model (5)	Model (6)
	Consultation (Log)	Follower (Log)	Consultation (Log)
Experience	0.136 *** (0.017)	0.124 *** (0.016)	0.031 ** (0.011)
Follower	-	-	0.877 *** (0.021)
Hospital	−0.192 * (0.093)	0.018 (0.089)	−0.208 *** (0.051)
Appointment	0.386 *** (0.043)	0.397 *** (0.039)	0.042 (0.028)
ClinicTitle	0.125 *** (0.030)	0.128 *** (0.028)	0.019 (0.019)
Recommendation	0.943 *** (0.047)	1.084 *** (0.041)	−0.006 (0.032)
Ratings	1.383 *** (0.062)	1.523 *** (0.058)	0.059 (0.057)
Price	0.002 *** (0.000)	0.002 *** (0.000)	−0.000 *** (0.000)
Duration	0.000 *** (0.000)	0.000 *** (0.000)	0.000 *** (0.000)
Views	0.000 ** (0.000)	0.000 *** (0.000)	0.000 * (0.000)
constant	0.550 ** (0.207)	0.309 (0.196)	0.236 * (0.120)
Observations	4119	4126	4119
R-squared	0.484	0.554	0.809
F test	328.72	391.58	1185.76

Robust t-statistics in parentheses. *** *p* < 0.01, ** *p* < 0.05, * *p* < 0.1.

**Table 8:** Robust test of main and moderating effect.

Variable	Model (7) Additional Controls	Model (8) Additional Controls	Model (9) Additional Controls
	Consultation	Consultation	Consultation
Experience	333.552 *** (65.154)	322.036 ** (68.569)	−84.468 (199.111)
Experience * Sentiment	-	-	463.396 ** (223.525)
Sentiment	-	−883.130 ** (373.069)	−950.800 *** (384.150)
Hospital	−705.318 * (327.104)	−696.068 (428.160)	−685.208 ** (429.377)
Appointment	278.410 * (147.160)	287.773 ** (125.591)	270.037 * (125.257)
ClinicTitle	271.199 ** (106.793)	298.735 *** (111.998)	299.905 *** (112.476)
Recommendation	3619.923 *** (181.546)	3634.657 *** (266.011)	3635.883 *** (265.970)
Ratings	981.783 *** (197.506)	952.558 *** (152.615)	951.255 *** (153.175)
Price	4.372 *** (0.662)	4.393 *** (0.856)	4.404 *** (0.858)
Duration	0.820 *** (0.058)	0.825 *** (0.070)	0.824 *** (0.070)
Views	0.001 *** (0.000)	0.001 ** (0.001)	0.001 *** (0.001)
Text Length	0.088 (0.148)	0.030 (0.208)	0.109 (0.718)
constant	−15,913.000 *** (789.766)	−15,247.790 *** (816.369)	−15,182.640 *** (816.797)
Observations	4126	4125	4126
F test	199.050	104.430	104.460
R-squared	0.326	0.328	0.328

Robust t-statistics in parentheses. *** *p* < 0.01, ** *p* < 0.05, * *p* < 0.1.

**Table 9:** Robust test of mediating effect.

Variable	Model (10) Additional Controls	Model (11) Additional Controls	Model (12) Additional Controls	Model (13) Using Negative Binomial Regression	Model (14) Using Negative Binomial Regression	Model (15) Using Negative Binomial Regression
	Consultation (Log)	Follower (Log)	Consultation (Log)	Consultation (Log)	Follower (Log)	Consultation (Log)
Experience	0.137 *** (0.017)	0.124 *** (0.016)	0.031 *** (0.011)	0.018 *** (0.002)	0.0169 *** (0.0022)	0.003 * (0.002)
Follower(log)	-	-	0.877 *** (0.021)	-	-	0.137 *** (0.003)
Hospital	−0.185 * (0.093)	0.021 (0.090)	−0.204 *** (0.051)	−0.027 * (0.012)	0.001 (0.012)	−0.026 *** (0.007)
Appointment	0.382 *** (0.043)	0.395 *** (0.039)	0.040 (0.028)	0.057 *** (0.006)	0.059 *** (0.006)	0.0111 ** (0.004)
ClinicTitle	0.119 *** (0.030)	0.125 *** (0.028)	0.015 (0.019)	0.018 *** (0.004)	0.018 *** (0.004)	0.002 (0.002)
Recommendation	0.944 *** (0.047)	1.084 *** (0.041)	−0.005 (0.032)	0.124 *** (0.006)	0.139 *** (0.005)	−0.024 *** (0.005)
Ratings	1.388 *** (0.063)	1.526 *** (0.058)	0.062 (0.057)	0.218 *** (0.010)	0.246 *** (0.010)	0.0253 ** (0.0086)
Price	0.001 *** (0.000)	0.002 *** (0.000)	0.000 ** (0.000)	0.000 *** (0.000)	0.000 *** (0.000)	0.000 *** (0.000)
Duration	0.000 *** (0.000)	0.000 *** (0.000)	0.000 *** (0.000)	0.000 *** (0.000)	0.000 *** (0.000)	0.000 *** (0.000)
Views	0.000 ** (0.000)	0.000 *** (0.000)	0.000 ** (0.000)	0.000 *** (0.000)	0.000 *** (0.000)	0.000 (0.000)
Text Length	0.0003 (0.0002)	0.0001 (0.0001)	0.0002 (0.0001)	-	-	-
constant	0.543 ** (0.207)	0.306 (0.196)	0.232 (0.119)	1.044 *** (0.028)	1.021 *** (0.027)	0.956 *** (0.017)
Observations	4119	4126	4119	4119	4126	4119
R-squared	0.4845	0.5537	0.8092	0.0423 (Pseudo)	0.0467 (Pseudo)	0.0741 (Pseudo)
F test	296.22	352.56	1078.94	2513.06	-	-

Robust t-statistics in parentheses. *** *p* < 0.01, ** *p* < 0.05, * *p* < 0.1.

## Data Availability

Data are contained within the article.

## Appendix A

This section includes two tables. The full list of keywords for the nine topics can be found in Table A1 (English version) or Table A2 (Chinese version).

**Table A1:** Probability distribution of topic keywords (English version).

Topic 1		Topic 2		Topic 3		Topic 4		Topic 5		Topic 6		Topic 7		Topic 8		Topic 9	
Keywords	Distribution Probability	Keywords	Distribution Probability	Keywords	Distribution Probability	Keywords	Distribution Probability	Keywords	Distribution Probability	Keywords	Distribution Probability	Keywords	Distribution Probability	Keywords	Distribution Probability	Keywords	Distribution Probability
Urology	0.03	Shanghai	0.022	Committee Member	0.029	Orthopedics	0.033	Surgery	0.027	Chief Physician	0.019	Skin	0.022	of	0.028	Surgery	0.031
Plastic	0.027	China	0.018	Branch	0.022	Stomatology	0.028	Treatment	0.027	Clinical	0.018	Dermatology	0.022	and	0.016	Treatment	0.03
Surgery	0.026	Tumor	0.014	Committee	0.021	Joint	0.023	Surgery	0.021	Work	0.017	Dermatology Department	0.021	in	0.012	Spine	0.02
Cosmetology	0.018	Committee Member	0.013	Professional	0.018	Surgery	0.018	Tumor	0.02	Disease	0.016	Treatment	0.016	Journal	0.011	Microinvasive	0.014
Plastic Surgery	0.017	Physician	0.011	Henan Province	0.016	Sports	0.018	Neurosurgery	0.019	Hospital	0.015	Cosmetology	0.01	the	0.008	Surgery	0.012
Treatment	0.015	Association	0.01	China	0.014	Injury	0.016	Disease	0.011	Graduate	0.013	Cicatrix	0.009	for	0.005	Technology	0.011
Tumor	0.012	Branch	0.01	Association	0.014	Maxillofacial	0.016	Endoscopy	0.01	Engaged in	0.012	Hemangioma	0.009	Chinese	0.005	Patient	0.009
Repair	0.012	Committee	0.01	Institution	0.014	Trauma	0.015	Microinvasive	0.01	Diagnosis and Treatment	0.01	Lupus Erythematosus	0.008	Research	0.005	Professor	0.008
Microinvasive	0.012	Clinical	0.01	Shandong Province	0.014	Repair	0.015	Chief Physician	0.01	Specializes in	0.01	Laser	0.008	with	0.005	Development	0.008
Laparoscopy	0.01	Surgery	0.009	Director Chairman	0.012	Arthroscopy	0.011	Laparoscopy	0.009	Medical School	0.01	Acne	0.008	Clinical	0.004	Domestic	0.008
Prostate Cancer	0.009	Research	0.009	Physician	0.011	Fracture	0.011	Work	0.009	Children	0.01	Dermatovenereology	0.008	Journal	0.004	Clinical	0.007
Specializes in	0.008	International	0.009	Medical Association	0.011	Treatment	0.01	Clinical	0.009	Affiliated	0.01	Rheumatism	0.008	cancer	0.004	Vascular	0.006
Reconstruction	0.008	Journal	0.009	Publication	0.01	Oral Medicine	0.01	Specializes in	0.008	Mentor	0.01	Psoriasis	0.007	Treatment	0.004	Interventional	0.006
Bladder	0.008	Medicine	0.009	Journal	0.009	Reconstruction	0.01	Resection	0.008	Research	0.009	Research	0.007	2008	0.004	Disease	0.006
Ureter	0.007	Hospital	0.008	Treatment	0.009	Knee Joint	0.009	Engaged in	0.007	Treatment	0.009	Systemic	0.007	Cancer	0.004	Work	0.006
Prostate	0.007	Professional	0.008	Chinese	0.008	Deformity	0.009	Research	0.007	Dr.	0.008	Hair	0.007	Zhang	0.004	Hospital	0.005
Deformity	0.006	National	0.008	Disease	0.008	Medical School	0.008	Hospital	0.007	Director	0.008	Specializes in	0.006	2010	0.003	Deformity	0.005
Chief Physician	0.006	Publication	0.008	Chinese Medical Association	0.008	Replacement	0.008	Diagnosis	0.006	Professor	0.007	Burn	0.006	Tumor	0.003	Neural	0.005
Facial	0.006	Professor	0.007	Hubei Province	0.008	Chief Physician	0.007	Liver Cancer	0.006	MD	0.007	Sexually Transmitted Disease	0.006	2013	0.003	End	0.004
Transplantation	0.006	Youth	0.007	More than	0.007	Implantation	0.007	Hepatobiliary	0.006	Gynecology	0.007	Facial	0.006	2015	0.003	Chief Physician	0.004

**Table A2:** Probability Distribution of Topic Keywords (Raw Text in Chinese).

Topic 1		Topic 2		Topic 3		Topic 4		Topic 5		Topic 6		Topic 7		Topic 8		Topic 9	
Keywords	Distribution Probability	Keywords	Distribution Probability	Keywords	Distribution Probability	Keywords	Distribution Probability	Keywords	Distribution Probability	Keywords	Distribution Probability	Keywords	Distribution Probability	Keywords	Distribution Probability	Keywords	Distribution Probability
泌尿外科	0.03	上海市	0.022	委员	0.029	骨科	0.033	手术	0.027	主任医师	0.019	皮肤	0.022	of	0.028	手术	0.031
整形	0.027	中国	0.018	分会	0.022	口腔	0.028	治疗	0.027	临床	0.018	皮肤病	0.022	and	0.016	治疗	0.03
手术	0.026	肿瘤	0.014	委员会	0.021	关节	0.023	外科	0.021	工作	0.017	皮肤科	0.021	in	0.012	脊柱	0.02
美容	0.018	委员	0.013	专业	0.018	外科	0.018	肿瘤	0.02	疾病	0.016	治疗	0.016	杂志	0.011	微创	0.014
整形外科	0.017	医师	0.011	河南省	0.016	运动	0.018	神经外科	0.019	医院	0.015	美容	0.01	the	0.008	外科	0.012
治疗	0.015	协会	0.01	中国	0.014	损伤	0.016	疾病	0.011	毕业	0.013	瘢痕	0.009	for	0.005	技术	0.011
肿瘤	0.012	分会	0.01	协会	0.014	颌面	0.016	内镜	0.01	从事	0.012	血管瘤	0.009	中华	0.005	患者	0.009
修复	0.012	委员会	0.01	学会	0.014	创伤	0.015	微创	0.01	诊治	0.01	红斑狼疮	0.008	研究	0.005	教授	0.008
微创	0.012	临床	0.01	山东省	0.014	修复	0.015	主任医师	0.01	擅长	0.01	激光	0.008	with	0.005	开展	0.008
腹腔镜	0.01	外科	0.009	主任委员	0.012	关节镜	0.011	腹腔镜	0.009	医学院	0.01	痤疮	0.008	临床	0.004	国内	0.008
前列腺癌	0.009	研究	0.009	医师	0.011	骨折	0.011	工作	0.009	儿童	0.01	皮肤性病	0.008	Journal	0.004	临床	0.007
擅长	0.008	国际	0.009	医学会	0.011	治疗	0.01	临床	0.009	附属	0.01	风湿	0.008	cancer	0.004	血管	0.006
重建	0.008	杂志	0.009	发表	0.01	口腔医学	0.01	擅长	0.008	导师	0.01	银屑病	0.007	治疗	0.004	介入	0.006
膀胱	0.008	医学	0.009	杂志	0.009	重建	0.01	切除	0.008	研究	0.009	研究	0.007	2008	0.004	疾病	0.006
输尿管	0.007	医院	0.008	治疗	0.009	膝关节	0.009	从事	0.007	治疗	0.009	系统性	0.007	Cancer	0.004	工作	0.006
前列腺	0.007	专业	0.008	中华	0.008	畸形	0.009	研究	0.007	博士	0.008	毛发	0.007	Zhang	0.004	医院	0.005
畸形	0.006	国家	0.008	疾病	0.008	医学院	0.008	医院	0.007	主任	0.008	擅长	0.006	2010	0.003	畸形	0.005
主任医师	0.006	发表	0.008	中华医学会	0.008	置换	0.008	诊断	0.006	教授	0.007	烧伤	0.006	肿瘤	0.003	神经	0.005
面部	0.006	教授	0.007	湖北省	0.008	主任医师	0.007	肝癌	0.006	医学博士	0.007	性病	0.006	2013	0.003	结束	0.004
移植	0.006	青年	0.007	余篇	0.007	种植	0.007	肝胆	0.006	妇科	0.007	面部	0.006	2015	0.003	主任医师	0.004

## References

[1] Schmitz A., Díaz-Martín A.M., Guillén J.Y. (2022). Modifying UTAUT2 for a cross-country comparison of telemedicine adoption. Comput. Hum. Behav..

[2] Mirzaei T., Esmaeilzadeh P. (2021). Engagement in online health communities: Channel expansion and social exchanges. Inf. Manag..

[3] Biancone P., Secinaro S., Marseglia R., Calandra D. (2023). E-health for the future. Managerial perspectives using a multiple case study approach. Technovation.

[4] Cheng X., Fu S., Sun J., Han Y., Shen J., Zarifis A. (2016). Investigating individual trust in semi-virtual collaboration of multicultural and unicultural teams. Comput. Hum. Behav..

[5] Wang X.H., Shi J.Y., Kong H.X. (2021). Online Health Information Seeking: A Review and Meta-Analysis. Health Commun..

[6] Zhao Y., Da J., Yan J. (2021). Detecting health misinformation in online health communities: Incorporating behavioral features into machine learning based approaches. Inf. Process. Manag..

[7] Taylor L.A., Nong P., Platt J. (2023). Fifty Years of Trust Research in Health Care: A Synthetic Review. Milbank Q..

[8] Kordzadeh N. (2019). Investigating bias in the online physician reviews published on healthcare organizations’ websites. Decis. Support Syst..

[9] Fan W.J., Zhou Q.Q., Qiu L.F., Kumar S. (2023). Should Doctors Open Online Consultation Services? An Empirical Investigation of Their Impact on Offline Appointments. Inf. Syst. Res..

[10] Chen L.T., Baird A., Straub D. (2020). A linguistic signaling model of social support exchange in online health communities. Decis. Support Syst..

[11] Liu S., Si G.S., Gao B.J. (2022). Which voice are you satisfied with? Understanding the physician-patient voice interactions on online health platforms. Decis. Support Syst..

[12] Zhou J., Zhang Q., Zhou S., Li X., Zhang X.M. (2023). Unintended Emotional Effects of Online Health Communities: A Text Mining-Supported Empirical Study. MIS Q..

[13] Wang L.A., Yan L., Zhou T.X., Guo X.T., Heim G.R. (2020). Understanding Physicians’ Online-Offline Behavior Dynamics: An Empirical Study. Inf. Syst. Res..

[14] Shoji M., Fujiwara A., Shimada A., Onda M. (2020). The relationship between community pharmacists’ social distance from and their confidence in interacting with patients with depression in Japan. Int. J. Clin. Pharm..

[15] Khurana S., Qiu L., Kumar S. (2019). When a Doctor Knows, It Shows: An Empirical Analysis of Doctors’ Responses in a Q&A Forum of an Online Healthcare Portal. Inf. Syst. Res..

[16] Liu S., Zhang M.Y., Gao B.J., Jiang G.Y. (2020). Physician voice characteristics and patient satisfaction in online health consultation. Inf. Manag..

[17] Gong Y.L., Wang H.W., Xia Q.W., Zheng L.J., Shi Y.X. (2021). Factors that determine a Patient’s willingness to physician selection in online healthcare communities: A trust theory perspective. Technol. Soc..

[18] Li Y., Song Y., Zhao W., Guo X., Ju X., Vogel D. (2019). Exploring the Role of Online Health Community Information in Patients’ Decisions to Switch from Online to Offline Medical Services. Int. J. Med. Inform..

[19] Wu J., Huang X., Sun P., Zhang X.F. (2022). What affects patients’ choice of consultant: An empirical study of online doctor consultation service. Electron. Commer. Res..

[20] Paige S.R., Krieger J.L., Stellefson M.L. (2017). The Influence of eHealth Literacy on Perceived Trust in Online Health Communication Channels and Sources. J. Health Commun..

[21] Parrillo V.N., Donoghue C. (2005). Updating the Bogardus social distance studies: A new national survey. Soc. Sci. J..

[22] Park G., Chung J.Y., Lee S.Y. (2024). Human vs. machine-like representation in chatbot mental health counseling: The serial mediation of psychological distance and trust on compliance intention. Curr. Psychol..

[23] He Y.Q., Tan X.M., Wang J.J., Wiley J., Huang Y.X., Ding H., Wang Q., Huang T.H., Sun M. (2024). Trust, discrimination and preference for shared decision-making in adolescents diagnosed with depression: Implications from Chinese mental health professionals. Patient Educ. Couns..

[24] Schnittker J. (2004). Social distance in the clinical encounter: Interactional and sociodemographic foundations for mistrust in physicians. Soc. Psychol. Q..

[25] Huang C.Z., Xie P., Liang W.S., Zhou A.B. (2024). “Kill the familiar effect”: The impact of anger on deceptive behavior. Curr. Psychol..

[26] He W.M., Qiu J.J., Chen Y.Y., Zhong Y.F. (2022). Gratitude Intervention Evokes Indebtedness: Moderated by Perceived Social Distance. Front. Psychol..

[27] Qiao W., Huang N., Yan Z. (2024). How to Translate Firm-Generated Content to Sales? Evidence from Online Healthcare Platforms. J. Manag. Inf. Syst..

[28] Zhou J.J., Kishore R., Amo L., Ye C. (2022). Description and demonstration signals as complements and substitutes in an online market for mental health care. MIS Q..

[29] Yuen K.F., Bin Saidi M.S., Bai X.W., Wang X.Q. (2021). Cruise transport service usage post COVID-19: The health belief model application. Transp. Policy.

[30] Xie J.H., Liu X., Zeng D.D.J., Fang X. (2022). Understanding Medication Nonadherence from Social Media: A Sentiment-Enriched Deep Learning Approach. MIS Q..

[31] Shen J., An B., Xu M., Gan D., Pan T. (2022). Internal or External Word-of-Mouth (WOM), Why Do Patients Choose Doctors on Online Medical Services (OMSs) Single Platform in China?. Int. J. Environ. Res. Public Health.

[32] Zhang W., Zhou F.Z., Fei Y.F. (2023). Repetitions in online doctor-patient communication: Frequency, functions, and reasons. Patient Educ. Couns..

[33] Goh K.Y., Heng C.S., Lin Z.J. (2013). Social Media Brand Community and Consumer Behavior: Quantifying the Relative Impact of User- and Marketer-Generated Content. Inf. Syst. Res..

[34] Duvall M., North F., Leasure W., Pecina J. (2021). Patient portal message characteristics and reported thoughts of self-harm and suicide: A retrospective cohort study. J. Telemed. Telecare.

[35] Caruana A. (2002). Service loyalty: The effects of service quality and the mediating role of customer satisfaction. Eur. J. Mark..

[36] Mosahab R., Mahamad O., Ramayah T. (2010). Service quality, customer satisfaction and loyalty: A test of mediation. Int. Bus. Res..

[37] Yan L., Tan Y. (2017). The Consensus Effect in Online Health-Care Communities. J. Manag. Inf. Syst..

[38] Li J., Tang J., Liu X., Ma L. (2019). How do users adopt health information from social media? The narrative paradigm perspective. Health Inf. Manag. J..

[39] Jin J.H., Yan X.B., Li Y.J., Li Y.M. (2016). How users adopt healthcare information: An empirical study of an online Q&A community. Int. J. Med. Inform..

[40] Meng F.B., Zhang X.F., Liu L.B., Ren C.C. (2021). Converting readers to patients? From free to paid knowledge-sharing in online health communities. Inf. Process. Manag..

[41] Yang X.J., Xi N.N., Gu D.X., Liang C.Y., Liu H., Tang H.R., Hamari J. (2024). Medical practice in gamified online communities: Longitudinal effects of gamification on doctor engagement. Inf. Manag..

[42] Guo S.S., Guo X.T., Fang Y.L., Vogel D. (2017). How Doctors Gain Social and Economic Returns in Online Health-Care Communities: A Professional Capital Perspective. J. Manag. Inf. Syst..

[43] Luhmann N. (2018). Trust and Power.

[44] Pizzutti C., Fernandes D. (2010). Effect of recovery efforts on consumer trust and loyalty in e-tail: A contingency model. Int. J. Electron. Commer..

[45] Hesse B.W., Nelson D.E., Kreps G.L., Croyle R.T., Arora N.K., Rimer B.K., Viswanath K. (2005). Trust and sources of health information—The impact of the Internet and its implications for health care providers: Findings from the first Health Information National Trends Survey. Arch. Intern. Med..

[46] Muda M., Hamzah M.I. (2021). Should I suggest this YouTube clip? The impact of UGC source credibility on eWOM and purchase intention. J. Res. Interact. Mark..

[47] Geng R.S., Chen J. (2021). The Influencing Mechanism of Interaction Quality of UGC on Consumers’ Purchase Intention—An Empirical Analysis. Front. Psychol..

[48] Gopichandran V., Chetlapalli S.K. (2013). Dimensions and determinants of trust in health care in resource poor settings—A qualitative exploration. PLoS ONE.

[49] Xu Y.X., Yang Z.S., Jiang H.Y., Sun P.Z. (2022). Research on patients’ willingness to conduct online health consultation from the perspective of web trust model. Front. Public Health.

[50] Yang H.L., Guo X.T., Wu T.S., Ju X.F. (2015). Exploring the effects of patient-generated and system-generated information on patients’ online search, evaluation and decision. Electron. Commer. Res. Appl..

[51] Dong W., Lei X.X., Liu Y.M. (2022). The Mediating Role of Patients’ Trust Between Web-Based Health Information Seeking and Patients’ Uncertainty in China: Cross-sectional Web-Based Survey. J. Med. Internet Res..

[52] Choi M., Choi Y., Bangura E., Kim D. (2024). ChatGPT or online review: Which is a better determinant of customers’ trust in Airbnb listings and stay intention?. Int. J. Hosp. Manag..

[53] Yang X. (2019). How perceived social distance and trust influence reciprocity expectations and eWOM sharing intention in social commerce. Ind. Manag. Data Syst..

[54] Walther J.B., Pingree S., Hawkins R.P., Buller D.B. (2005). Attributes of interactive online health information systems. J. Med. Internet Res..

[55] Yang H.L., Du H.S., Shang W. (2021). Understanding the influence of professional status and service feedback on patients’ doctor choice in online healthcare markets. Internet Res..

[56] Wu H., Deng Z.H., Wang B., Gupta S. (2021). How does service price influence patients’ decisions? An examination of the free-market pricing mechanism in online health communities. Electron. Mark..

[57] Liu X.C., Xu Z., Yu X.T., Oda T. (2022). Using Telemedicine during the COVID-19 Pandemic: How Service Quality Affects Patients’ Consultation. Int. J. Environ. Res. Public Health.

[58] Liu H., Zhang Y., Li Y.L., Albright K. (2023). Better interaction performance attracts more chronic patients? Evidence from an online health platform. Inf. Process. Manag..

[59] Jiang F., Liu Y., Hu J., Chen X. (2022). Understanding Health Empowerment From the Perspective of Information Processing: Questionnaire Study. J. Med. Internet Res..

[60] Blei D.M., Ng A.Y., Jordan M. (2003). Latent dirichlet allocation. Adv. Neural Inf. Process. Syst..

[61] Jelodar H., Wang Y., Yuan C., Feng X., Jiang X., Li Y., Zhao L. (2019). Latent Dirichlet allocation (LDA) and topic modeling: Models, applications, a survey. Multimed. Tools Appl..

[62] Sievert C., Shirley K. LDAvis: A method for visualizing and interpreting topics. Proceedings of the Workshop on Interactive Language Learning, Visualization, and Interfaces.

[63] Chuang J., Manning C.D., Heer J. Termite: Visualization techniques for assessing textual topic models. Proceedings of the International Working Conference On Advanced Visual Interfaces.

[64] Hayashi F. (2011). Econometrics.

[65] Agostinelli C.J. (2002). Robust stepwise regression. J. Appl. Stat..

[66] Andrews D.W. (1994). Empirical process methods in econometrics. Handbook of Econometrics.

